# Dataset of seized wildlife and their intended uses

**DOI:** 10.1016/j.dib.2021.107531

**Published:** 2021-10-30

**Authors:** Oliver C. Stringham, Stephanie Moncayo, Eilish Thomas, Sarah Heinrich, Adam Toomes, Jacob Maher, Katherine G.W. Hill, Lewis Mitchell, Joshua V. Ross, Chris R. Shepherd, Phillip Cassey

**Affiliations:** a Invasion Science & Wildlife Ecology Lab, University of Adelaide, SA 5005, Australia; b School of Mathematical Sciences, University of Adelaide, SA 5005, Australia; c Monitor Conservation Research Society, Big Lake Ranch, BC, Canada

**Keywords:** CITES, Dark web, Illegal wildlife trade, Internet, LEMIS, Social media, Wildlife products, Wildlife seizures

## Abstract

The illegal wildlife trade (IWT) threatens conservation and biosecurity efforts. The Internet has greatly facilitated the trade of wildlife, and researchers have increasingly examined the Internet to uncover illegal trade. However, most efforts to locate illegal trade on the Internet are targeted to one or few taxa or products. Large-scale efforts to find illegal wildlife on the Internet (e-commerce, social media, dark web) may be facilitated by a systematic compilation of illegally traded wildlife taxa and their uses. Here, we provide such a dataset. We used seizure records from three global wildlife trade databases to compile the identity of seized taxa along with their intended usage (i.e., use-type). Our dataset includes c. 4.9k distinct taxa representing c. 3.3k species and contains c. 11k taxa-use combinations from 110 unique use-types. Further, we acquired over 45k common names for seized taxa from over 100 languages. Our dataset can be used to conduct large-scale broad searches of the Internet to find illegally traded wildlife. Further, our dataset can be filtered for more targeted searches of specific taxa or derived products.

## Specifications Table

**Table:** 

Subject	Ecology
Specific subject area	Illegal wildlife trade
Type of data	Table
How data were acquired	This dataset is a compilation and curation of three global wildlife trade databases that include seizures of illegally traded wildlife. The datasets are: (i) The TRAFFIC International Wildlife Trade Portal; (ii) CITES (Convention on International Trade in Endangered Species of Wild Fauna and Flora) trade database; and (iii) LEMIS (Law Enforcement Management Information System from the United States Fish and Wildlife Services) trade database. TRAFFIC and CITES databases are openly accessible and LEMIS data were recieved from a Freedom of Information Act request to the United States government. In addition, we obtained common names and taxonomic information of seized taxa from the GBIF (Global Biodiversity Information Facility) taxonomic database.
Data format	Raw and filtered
Parameters for data collection	This dataset comprises of the identity of the taxa involved in wildlife seizures, along with their intended use-type (e.g., live, medicine, meat), dated between January 1, 2010 to December 31, 2019.
Description of data collection and data source location	We accessed wildlife seizures from the following global wildlife trade databases: (i) The TRAFFIC International Wildlife Trade Portal, using their website (https://www.wildlifetradeportal.org/). (ii) CITES trade database, using their website (https://trade.cites.org/) (iii) LEMIS trade database, from Freedom of Information Act requests to the United States government. We resolved taxonomic names using the GBIF (https://www.gbif.org/) taxonomic database. We accessed and collected upstream taxonomic information (e.g., Family, Order, Class) and common names of seized wildlife from GBIF.
Data accessibility	Data is hosted in a public repository. Repository name: figshare Direct URL to data: https://figshare.com/articles/dataset/Dataset_of_seized_wildlife_and_their_intended_uses/14914773

## Value of the Data

• The illegal wildlife trade (IWT) presents a suite of biosecurity, welfare, and conservation concerns. Increasingly, IWT occurs on the Internet and researchers are seeking ways to find and quantify IWT. Our dataset provides a comprehensive list of taxa involved in IWT (c. 3.3k species), their common names, and their intended usage. This dataset can be used to generate keywords to search the Internet (e-commerce marketplaces, social media, and dark web) to locate IWT.
• Resources and tools that assist in the detection of IWT are beneficial to researchers, law enforcement, and organizations interested in finding, and combatting, IWT. This dataset will be useful for researchers in academic institutions, government agencies, and non-profit organisations for searching and locating IWT occurring on the Internet. Ultimately, if IWT is found on the Internet, this information can assist law enforcement to find and prosecute suspects and help organizations efficiently target consumer-demand reduction campaigns, as well as gauge the extent of IWT on specific internet platforms.
• Our dataset will be most useful for non-targeted sweeps of the Internet for IWT (i.e., looking for any illegal trade, not of a single species or product). However, our dataset can be filtered to create more targeted searches (e.g., all species of birds whose feathers were seized). Further, our dataset can be used to explore taxonomic trends and biases in wildlife seizures and provide a baseline for comparisons with future analogous data.

## 1 Data Description

**Fig. 1:**
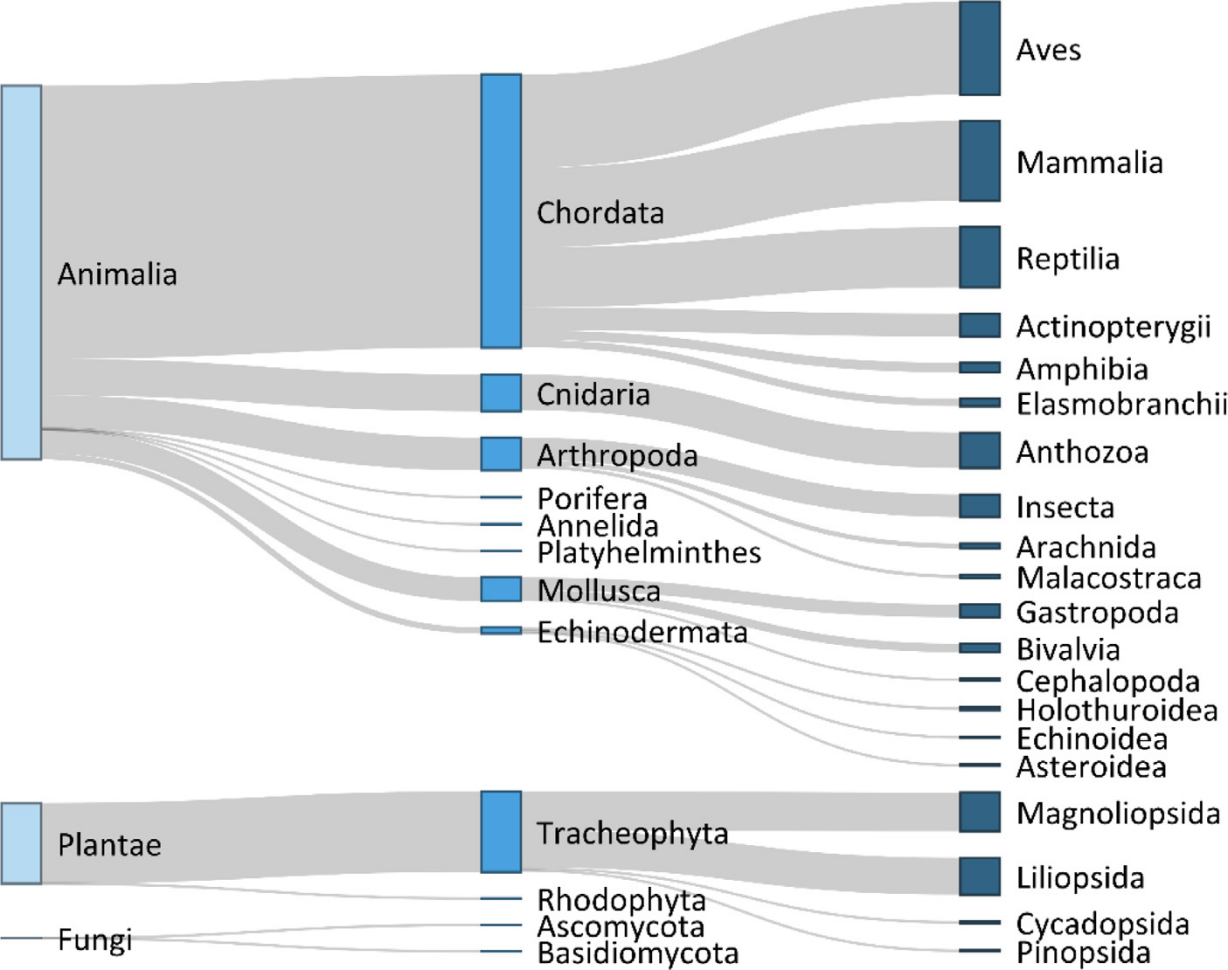
Diversity of wildlife taxa illegally traded as reported in the databases of CITES, LEMIS and TRAFFIC. Widths of bars correspond to the number of taxa in each taxonomic group. The leftmost column displays the taxonomic kingdom, the middle column displays the phylum, and right column displays the order. Taxonomic orders with less than 10 taxa are not displayed.

The presented data covers the illegal trade (i.e., wildlife seizures; [1]) of 4,899 distinct taxa across three kingdoms (Fig. 1). The most diverse taxonomic kingdom was Animalia (n = 4,026 taxa), followed by Plantae (n = 871), then Fungi (n = 2). We identified c. 71% of the taxa to the level of species (or more specific) and c. 95% of taxa to the level of genus (Table 1). In total, our dataset represents 3,361 species. We used GBIF (Global Biodiversity Information Facility) to standardize taxonomy and obtain upstream taxonomic information [2].

**Table 1:** Taxa in this dataset are stratified by their taxonomic rank. Each wildlife seizure record is accompanied by a name for the taxon that was seized. For each record, we identified the taxon to the most specific taxonomic rank possible. Thus, the ‘Number of taxa’ column represents the number of taxa for the specified rank only, and not the total number of taxa identified to that rank. For example, there were 3,340 taxa identified to the rank of species, however, 159 taxa were identified as more specific than species (variety and subspecies). Of those 159 taxa, 21 had not been recorded at the species level as seized, thus, 3,361 species (3,340 + 21) are present in this dataset.

Rank of taxa	Number of taxa	Cumulative number of taxa	Cumulative proportion of taxa
variety	2	2	0.000
subspecies	157	159	0.032
species	3,340	3,499	0.714
genus	1,147	4,646	0.948
family	173	4,819	0.984
order	41	4,860	0.992
class	20	4,880	0.996
phylum	8	4,888	0.998
kingdom	2	4,890	0.998
hybrid	9	4,899	1.000

We standardized biological and resource use-types (e.g., “ivory”, “meat”, “live”) given by the three trade databases (TRAFFIC, CITES, LEMIS), resulting in 110 ‘standardized’ use-types. We further categorized these standardized use-types into 4 main categories (live, dead/raw, processed/derived, and unspecified) and 40 sub-categories for data summary purposes (Table 2; Table 3; Table S1). The most diverse main categories of seizures (measured by the number of taxa) were “dead/raw”, followed by “live”, then “processed/derived” (Table 2). The most diverse sub-categories were: “live organisms or parts”, “dead organisms (whole body)”, and bone or bone-like body parts (Table 3). The most diverse standardized use-type was “live”, where over 2,127 distinct taxa were seized whole and alive (e.g., for the pet and ornamental plant trade; [3,4]), followed by seizures of dead wildlife.

**Table 2:** Main categories of wildlife seizures and number of unique taxa belonging to each category. The same taxa may be recorded as seized under more than one use-type or use-type category (e.g., live python and python skin). Thus, the ‘Number of taxa’ column is greater than the total number of taxa in this dataset.

Use-type main category	Description	Number of taxa
dead/raw	The dead/raw use-type category corresponds to dead whole organisms and unprocessed parts of dead organisms. This category includes the following: dead whole animals, taxidermized animals, animal trophies, fur, skin, bones, scales, horns, tusks, extracts (e.g., bile), organs, spines, wood, and timber.	6,133
live	The live use-type category represents organisms seized while alive. This category includes the following: live animals or plants, eggs, coral, and live plant parts.	2,285
processed/derived	The processed/derived use-type category represents derived or processed wildlife. This category includes the following: alcohol, processed food, horn and ivory carvings, jewellery, powder, leather, and clothing.	1,943
unspecified	The unspecified use-type category was used when a database did not specify the use-type of the taxa that was seized.	465

**Table 3:** Use-type subcategories with number of taxa in each subcategory. The top 20 (of 40) subcategories are shown. The definitions of each use-type subcategory can be found in Table S1.

Use-type main category	Use-type subcategory	Number of taxa
live	live	2,173
dead/raw	dead (whole animal)	1,642
dead/raw	animal parts (bone or bone-like)	623
dead/raw	animal fibers	445
unspecified	unspecified	426
dead/raw	skin/leather (raw)	414
dead/raw	food (raw)	389
dead/raw	taxidermy	381
dead/raw	animal parts (fleshy)	297
processed/derived	clothing	262
processed/derived	skin/leather (products)	258
dead/raw	shells (raw)	233
processed/derived	medicine	216
processed/derived	derivative	209
processed/derived	jewellery & personal ornaments	203
dead/raw	coral (dead)	174
dead/raw	wood/timber	163
dead/raw	extract	147
processed/derived	carvings/engravings	126
processed/derived	shells (product)	123

**Fig. 2:**
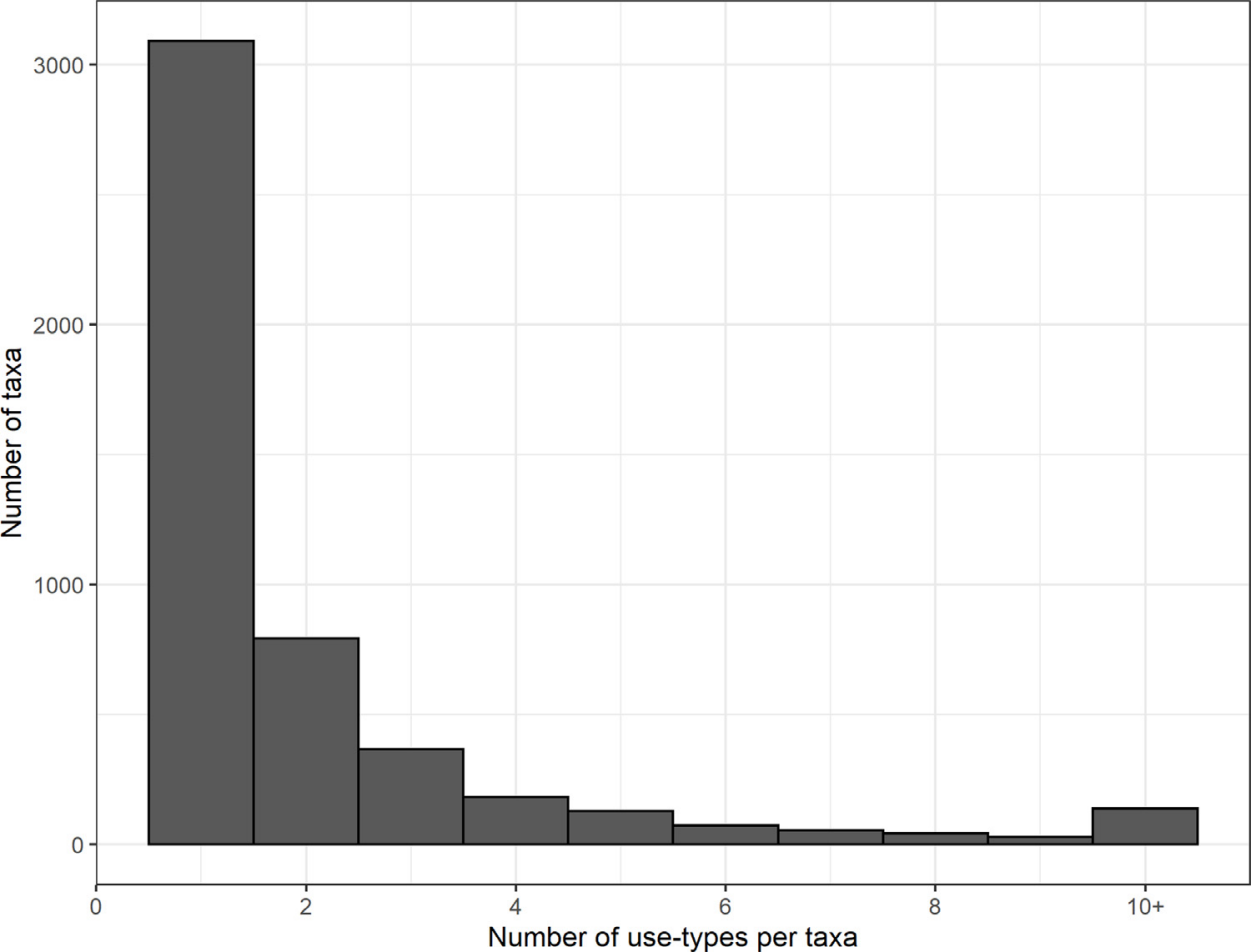
The number of use-type designations per taxa. There were 139 taxa (c. 3%) with 10 or more use-types.

**Fig. 3:**
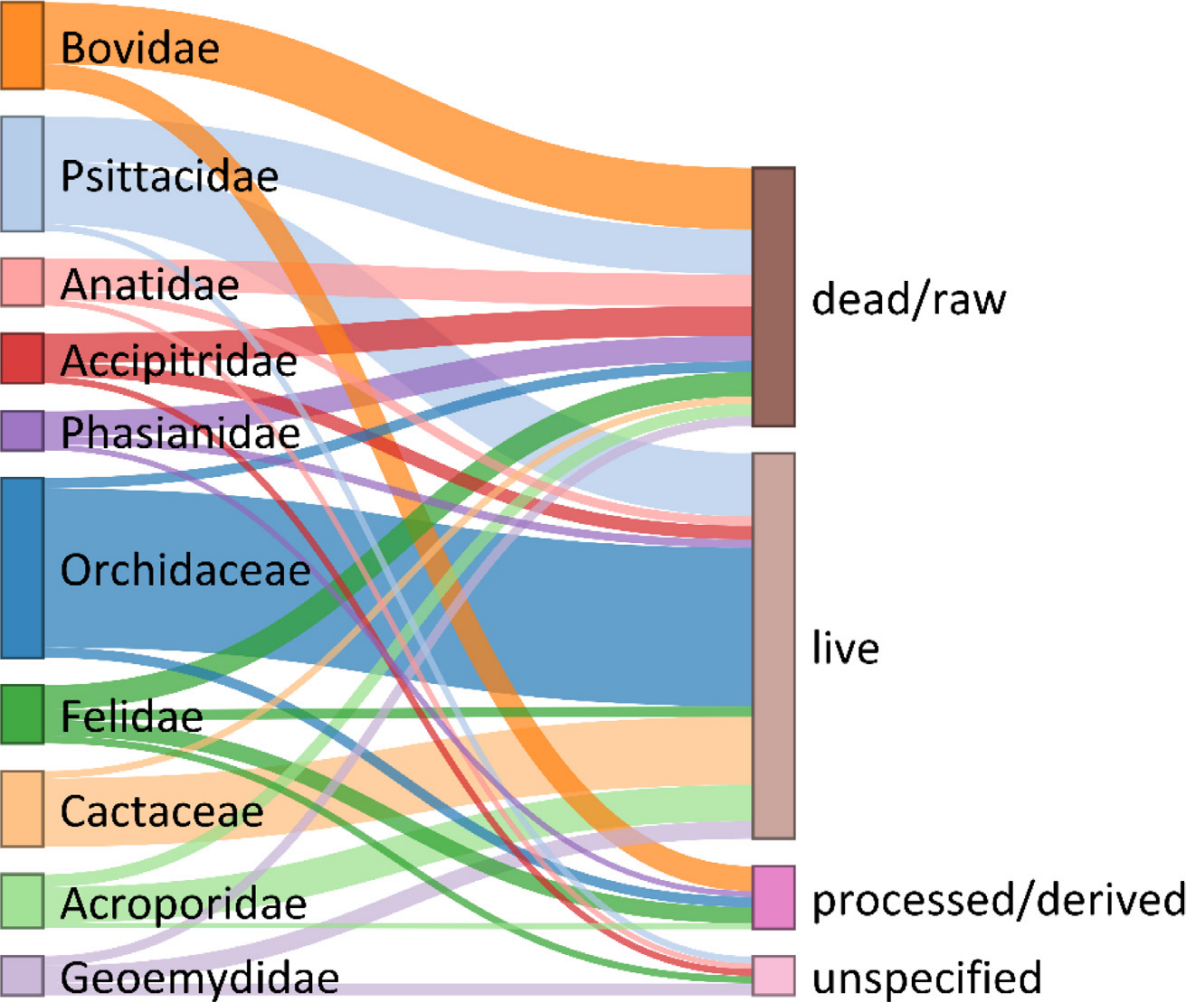
Use-taxa combinations at the family taxonomic level. The top 10 families (by number of use-taxa combinations) and the four main categories are displayed. Line thickness represents the number of unique taxa within each family that belong to a corresponding use-type main category (e.g., number of taxa in Orchidaceae that were seized as ‘live’; n = 325).

In total, we compiled 10,745 unique taxa-use combinations. We define a taxa-use combination as a unique combination of one taxa and one standardized use-type (e.g., bear claw). For taxa identifiable to the species level, we compiled 7,183 species-use combinations. We recorded multiple use-types for c. 37% of all seized taxa (n = 1,807 taxa); however, the majority of taxa had one use-type (Fig. 2). The most common taxa-use combinations, at the rank of taxonomic family, were: live seizures of orchids (Orchidaceae, n = 325 taxa); live seizures of cacti (Cactaceae, n = 136) and live seizures of Neotropical and Afrotropical parrots (Psittacidae, n = 126) (Fig. 3). The single species with the most use-types was the tiger (*Panthera tigris*), which had 35 distinct use-types (e.g., bone, skin, genitalia; Table 4).

**Table 4:** Species with the most recorded number of use-types (top 10 species shown).

Scientific name	Common name	Number of use-types
*Panthera tigris*	Tiger	35
*Loxodonta africana*	African bush elephant	33
*Panthera pardus*	Leopard	29
*Panthera leo*	Lion	26
*Elephas maximus*	Asian elephant	25
*Ursus arctos*	Brown bear	25
*Cervus elaphus*	Red deer	24
*Crocodylus niloticus*	Nile crocodile	24
*Alligator mississippiensis*	American alligator	22
*Odobenus rosmarus*	Walrus	21
*Ursus americanus*	American black bear	21

We retrieved the common names for each resolved taxa from GBIF, along with the common names associated with each taxa's upstream taxonomy. In total, we recorded 8,832 common names in the English language, and a further 37,507 common names in 125 other languages (Table 5). However, we found only 13 languages with over 1,000 common names. For approximately 7% of the common names returned, GBIF did not provide what language the common name was (i.e., the language field was left blank; n = 3,734 names).

**Table 5:** Common names retrieved from GBIF (Global Biodiversity Information Facility) stratified by language.

Language	Number of common names	Proportion	Cumulative proportion
English	8,832	0.18	0.18
German	3,036	0.06	0.24
Spanish	3,008	0.06	0.30
French	2,535	0.05	0.35
Danish	1,970	0.04	0.39
Swedish	1,916	0.04	0.43
Chinese	1,857	0.04	0.46
Japanese	1,826	0.04	0.50
Portuguese	1,802	0.04	0.53
Dutch/Flemish	1,544	0.03	0.57
Bokmål	1,259	0.03	0.59
Italian	1,142	0.02	0.61
Russian	1,084	0.02	0.64
Polish	954	0.02	0.65
Norwegian	928	0.02	0.67
Finnish	922	0.02	0.69
Estonian	850	0.02	0.71
Lithuanian	846	0.02	0.73
Czech	830	0.02	0.74
Other languages	9,198	0.18	0.93
No language specified	3,734	0.07	1.00

Two seizure databases (TRAFFIC and LEMIS) provided common names and one database (LEMIS) provided ‘generic’ names. A ‘generic’ name is either an alternative common name, regional name, trade name (a name used by traders but not the scientific and/or citizen science community), or the name of the family, order, or class of the taxa of interest. For example, Elephant would be a ‘generic’ name for the African bush elephant (*Loxodonta africana*). In total, we recorded 2,251 common names and 881 generic names from the trade databases (predominantly English language names). Of those, 727 common names and 247 ‘generic’ names were not found in the common names collected from GBIF.

For each standardized use-type, we assigned ‘Internet friendly’ search terms that are relevant synonyms of each use-type. In total, we derived 304 search terms, where each use-type contained from zero (i.e., for “live” and “dead” seizures without a specified use) to eight use-specific search words, with a median of 2 search words per standardized use-type.

We provide the above-described data in five tables that can be found in a public data repository (https://figshare.com/articles/dataset/Dataset_of_seized_wildlife_and_their_intended_uses/14914773). The tables included are as follows: (i) taxa-use combinations, named “data/01_taxa_use_combos.csv” in the data repository, (ii) taxonomic key of GBIF taxonomy, named “data/02_gbif_taxonomic_key.csv”, (iii) common names provided by GBIF, named “data/03_gbif_common_names.csv”, (iv) common names provided by LEMIS and TRAFFIC, named “data/04_db_generic_common_names.csv”, and, (v) ‘Internet friendly’ search words associated with each use-type, named “data/05_use_search_words.csv”. We provide metadata describing each table and their fields in the data repository. These tables contain keys that allow for their combination (e.g., join or merge) to obtain a list of searchable keyword phrases tailored to one's requirement. For example, one can obtain a list of bird species that were seized as feathers, along with their common names. We provide R code, in the data repository, to demonstrate how to combine these datasets to obtain a list of searchable phrases.

## 2 Experimental Design, Materials and Methods

Our goal was to compile a comprehensive list of the wildlife taxa involved in the IWT (i.e., wildlife seizures) along with the purpose for which they were being traded (i.e., use-type). We chose to restrict our search to contemporary IWT (since 2010), because we intend this dataset to be used for searching the Internet, where trading wildlife is a relatively recent phenomenon [5,6].

### 2.1 Data sources

We compiled wildlife seizure records from three major wildlife trade databases: (i) TRAFFIC's Wildlife Trade Portal (TRAFFIC, with permission; https://www.wildlifetradeportal.org/), (ii) Convention on International Trade in Endangered Species of Wild Fauna and Flora trade database (CITES; https://trade.cites.org/), and (iii) United States Fish and Wildlife Service's Law Enforcement Management Information System (LEMIS; see [7] for more information on LEMIS). We obtained LEMIS through a Freedom of Information Act request to the United States government. Both TRAFFIC's and CITES databases are openly accessible. We restricted the date of wildlife seizures from 1 January 2010 to 31 December 2019, except for LEMIS, where our records stop at 31 December 2018. For all databases, we only extracted records labelled as seizures. For the TRAFFIC database, we extracted all records of ‘live’ or ‘dead’ seizures and the first 300 records (chronologically) from all other use-type categories.

While these three databases are among the most comprehensive wildlife trade databases available, we note that each database has biases and limitations. TRAFFIC's database is largely derived from open source data (e.g., media and government press releases) and thus is not a comprehensive record of wildlife seizures. Further, TRAFFIC's records tend to be taxonomically biased towards charismatic species (e.g., [8]) and is spatially biased towards countries where TRAFFIC staff are based and collecting data from. The CITES trade database primarily contains legal trade records, but only a subset of participating countries have reported seizure records through the database. Even the countries that do report seizures in the CITES trade database may not do so in a consistent manner and, thus, there is no way to distinguish between seizures of illegal wildlife and legal trade in previously confiscated wildlife [9,10]. The LEMIS database is taxonomically comprehensive but only involves seizures of wildlife that are linked to the United States of America [11].

### 2.2 Use-type cleaning and curation

Each seizure record gathered from the trade databases contained the use-type (i.e., intended usage of the wildlife). However, each trade database used slightly different words for the use-types. Thus, we standardized and consolidated the use-types between the three trade databases. Further, we provided ‘Internet-friendly’ search words associated with each use-type. These search words were either alternative names for the use-types used in one of the trade databases or synonyms of the use-type. For example, the search words we generated for the use-type “foetus” are “foetus”, “fetus”, “placenta”, and “embryo”. For the “live” and “dead” use-types, we did not assign any search words. We did not record the number of incidences for each taxa-use combination because there are likely duplicated seizure records between the three trade databases.

### 2.3 Taxa resolution

We resolved the taxonomic names from each trade database to the Global Biodiversity Information Facility taxonomic database (GBIF; [2]). We automated the taxa resolution process using the R package *taxize* [12]. We manually resolved each taxa that was not matched through automation. We obtained upstream taxonomic information from GBIF (e.g., family, order, class, etc.).

### 2.4 Common names

We collected the common names (i.e., vernacular names) from GBIF, for each taxa resolved to GBIF along with the common names for each upstream taxonomic unit. For example, for *Psittacus erithacus*, we retrieved the species vernacular name (African Gray Parrot), the family common name (African & New World Parrots), the order common name (Parrots), and the class common name (Bird). In some instances, GBIF provided multiple common names per taxonomic unit (i.e., multiple species common names). For each English common name, we took the singular form (e.g., bears was converted to bear), using the R package *pluralize* [13]. Further, we collected common names in other languages where available, from GBIF. In addition, two databases (TRAFFIC and LEMIS) provided common or ‘generic’ names (e.g., Parrot) of the taxa seized, and we included these names, as a separate table, in our dataset.

### 2.5 Software Used

We performed all data processing, analysis, and summaries in R (v. 3.6.3; [14]). We automated taxa resolution using the `get_gbif_id` function in the *taxize* package (v. 0.9.95.91; [12]). We automated the collection of upstream taxonomic information from GBIF using the `classification` function from the *taxize* package. We automated the collection of vernacular names from GBIF using the `name_usage` function from *rgbif* package (v. 3.3.0 [15]). We used the *tidyverse* ecosystem of packages for general data processing, analysis, and plotting (v. 1.3.0 [16]).

## Ethics Statement

Ethics statements are not required for the presented data. Our work did not involve human subjects, animal experiments, nor collect data from social media platforms.

## CRediT Author Statement

**Oliver C. Stringham:** Conceptualization, Methodology, Software, Investigation, Writing - Original Draft, Writing - Review & Editing, Visualization, Supervision **Stephanie Moncayo:** Investigation, Data Curation, Writing - Review & Editing **Eilish Thomas:** Investigation, Data curation, Writing - Review & editing **Sarah Heinrich:** Investigation, Data Curation, Writing - Review & Editing **Adam Toomes:** Investigation, Data Curation, Writing - Review & Editing **Jacob Maher:** Investigation, Data Curation, Writing - Review & Editing **Katherine G.W. Hill:** Investigation, Data Curation, Writing - review & editing **Lewis Mitchell:** Funding acquisition, Writing - review & editing **Joshua V. Ross:** Funding acquisition, Writing - review & editing **Chris R. Shepherd:** Writing - Review & Editing **Phillip Cassey:** Supervision, Funding acquisition, Writing - review & editing

## Declaration of Competing Interest

The authors declare that they have no known competing financial interests or personal relationships that could have appeared to influence the work reported in this paper.

## Data Availability

Dataset of seized wildlife and their intended uses (Original data) (figshare).

## Appendix Supplementary materials

Image, application 1
