# Supplementary material for: Dataset of seized wildlife and their intended uses
Source: Data Brief. 2021 Oct 30;39:107531. doi: 10.1016/j.dib.2021.107531 (PMC8579131; doi:10.1016/j.dib.2021.107531)
Supplement: Supplementary file 1 [file mmc1.docx]

**Dataset of seized wildlife and their intended uses**

Oliver C. Stringham, Stephanie Moncayo, Eilish Thomas, Sarah Heinrich, Adam Toomes, Jacob Maher, Katherine G.W. Hill, Lewis Mitchell, Joshua V. Ross, Chris R. Shepherd, Phillip Cassey

Table S1. Definitions of subcategories we used to categorize use types.

| **Main Category** | **Subcategory** | **Definition** |
| --- | --- | --- |
| processed/derived | accessories | Small non-jewellery items made of unspecified material, including: belts, belt buckles, bags, handbags, suitcases, cups, and knives. Does not include animal fiber products or leather products. |
| processed/derived | alcohols | Alcoholic beverages derived from wildlife, such as tiger bone wine. |
| dead/raw | animal fibers | Unprocessed furs (without skin), hairs (individual strands) and feathers, including those found in jewellery and other commodities. |
| dead/raw | animal parts (bone or bone-like) | Bony or bone-like animal parts, such as: bones, skulls, skeletons, horns, tusks, ivory, teeth, rostrums, casques, baleen. |
| dead/raw | animal parts (fleshy) | Animal parts with flesh and/or skin intact such as: feet, claws, tails, genitalia. |
| dead/raw | animal parts (unspecified) | Unspecified animal parts. |
| processed/derived | bone carvings/products | Bone carvings or related products, including jewellery made of bone. |
| processed/derived | carvings/engravings | Hard, processed carvings/engravings and related products that have no specified material. Does not include carvings or engravings derived from coral, ivory, horn, bone, wood or animal fiber, which all have own sub-categories. |
| processed/derived | clothing | Clothes and garments, including shoes, made of other than leather or plant fibers. |
| dead/raw | coral (dead) | Raw coral. |
| live | coral (live) | Live coral. |
| processed/derived | coral product | Products made or processed out of coral or coral stone, including coral figurines, sculptures and jewellery. |
| processed/derived | cosmetics | Cosmetic products derived from wildlife material. |
| dead/raw | dead (whole animal) | Dead bodies of animals, including animals that died during transport and including (but not limited to) specimens for museums and zoos. Note that animals that died during transport are recorded as dead, though they would have been recorded as 'live' if they had survived. |
| processed/derived | derivative | Products that have been processed/derived from the raw form, including oils, creams and balms (which are derived from rendered fat), gelatines, glues/adhesives, food supplements, pills and tablets and any chemical substances that require reconstitution. |
| dead/raw | egg (dead) | Non-live eggs, including eggshells and dead eggs. Caviar and roe are not included. |
| live | egg (live) | Eggs from birds or reptiles assumed to be alive. |
| dead/raw | extract | Unprocessed raw organic compounds that have been directly or indirectly extracted from an animal or plant. For example, civet, musk, ambergris, bile, venom, poison, semen, bezoar. |
| dead/raw | foetus/embryo | Any dead foetus or embryo that is contained ex situ from an egg. |
| processed/derived | food (processed) | Food products for human consumption, including broths, salep, soups. |
| dead/raw | food (raw) | Edible food, including meat and caviar. |
| live | fry (fish) | Juvenile fish: fry, fingerlings, and glass eels. |
| processed/derived | horn carvings/products | Hard, processed products derived from horn or antler. |
| processed/derived | ivory carvings/products | Hard, processed products derived from ivory, including carvings, engravings, jewellery and piano keys. |
| processed/derived | jewellery & personal ornaments | Jewellery and jewellery pieces, personal ornaments (amulets, talismans, etc.) or personal ornaments (figurines, statues, sculptures), often with no material specified. Does not include jewellery or ornaments derived from coral, ivory, horn, bone, wood or animal fiber, which all have own sub-categories. |
| live | live | Live animal or live plant (includes whole plants, seedlings, seeds, bulbs). Does not include live eggs, live coral, or live fry. |
| processed/derived | medicine | Medicinal products with no additional information regarding the type of product or what it was derived from. |
| dead/raw | nests | Bird nests and nest-derived products. |
| dead/raw | organs & tissues | Raw, unprocessed internal organs and tissues, such as: hearts, brains, eyes, fat, gall bladders. |
| dead/raw | plant fibers | High-cellulose plant material, such as: rope, fibers, tennis racquet strings. Does not include wood and timber. |
| dead/raw | plant parts | Flowers, stems, leaves, and fruit, including dried parts. Does not include live plants, bulbs, seeds, wood or timber. |
| processed/derived | powder | Wildlife in powdered form, includes those derived from horn, plant material, corals etc. |
| dead/raw | scales/spines | Raw or unprocessed: pangolin scales, reptiles scales, fish scales, quills, and spines (e.g., relating to porcupines). |
| processed/derived | shells (product) | Shells of seashells and turtles/tortoises that have been processed into a product. |
| dead/raw | shells (raw) | Seashell or turtle shells. |
| processed/derived | skin/leather (products) | Products made or processed out of leather, including 'leather', tanned skin, large and small leather products or items, shoes, skins, rugs, garments and trims. |
| dead/raw | skin/leather (raw) | Raw animal hides or skin pieces. |
| dead/raw | taxidermy | Taxidermized animals, including trophies. |
| unspecified | unspecified | Raw or processed products with no specified use-type or material. |
| processed/derived | wood product | Products made or processed out of wood, including: wooden figurines, sculptures, jewellery, furniture and charcoal. Excludes raw wood, which has its own subcategory. |
| dead/raw | wood/timber | Wood and timber, including: wood chips, wood veneers, sawn wood, plywood, and roundwood |
